# Do Heads Grow with Advanced Age?

**Published:** 1890-05

**Authors:** 


### Do Heads Grow with Advanced Age?

Some amusing letters have appeared in a daily contemporary in regard to an alleged steady increase in the size of Mr. Gladstone's head, which, it is said,is rendered manifest by a progressive enlargement in the size of the hat required to cover it. The correspondence exhibits an extraordinary ignorance of well ascertained facts; for if there is one thing which would be acknowledged by all anatomists and physiologists, it is that the nervous system, like other parts of the body, undergoes atrophy with advancing age—an atrophy that pervades every tissue, and is as apparent in the thinning of the vocal cords that alters the voice to " childish treble," as in the shrunk shanks for which the "youthful hose, well saved, are a world too wide." No reason can be assigned why the brain should escape the general change that affects the digestive and the circulatory systems alike. Its attributes and faculties attain their highest excellence at or before mid-age, and from that time forth exhibit only a steady decline. To compare Mr. Gladstone with Napoleon, respecting whom a similar story is related, is absurd. The head of Napoleon may have grown between 20 and 45, because his brain was greatly exercised during the last ten years of the past century and the first ten of the present; but no calls have been made on Mr. Gladstone of late years at all comparable to the strain on the mental and bodily powers of the French Emperor during that eventful period. The ossification of the sutures of the cranium practically prevents increase of the volume of the brain in advanced life; and, even granting some slight increase, such increase would be compensated for by attenuation of the cranial bones which is well known to occur in old age. A change in form, there may be, but none in size.—*The Lancet.*

Invention of the Microscope.—The third centenary of the invention of the microscope will be celebrated this year at Antwerp, where an historical exhibition of microscopes will be held, and public demonstrations will be given of the structure of the instrument and of its development from its first beginnings to its present form.

